# The correlation between UDP-glucuronosyltransferase polymorphisms and environmental endocrine disruptors levels in polycystic ovary syndrome patients

**DOI:** 10.1097/MD.0000000000019444

**Published:** 2020-03-13

**Authors:** Yunyao Luo, Ying Nie, Lu Tang, Charles C. Xu, Liangzhi Xu

**Affiliations:** a Department of Obstetrics and Gynecology, West China Second University Hospital, Sichuan University; b Reproductive Endocrinology and Regulation Laboratory, West China Second University Hospital, Sichuan University, Chengdu, Sichuan Province; c The Joint Laboratory for Reproductive Medicine of Sichuan University, The Chinese University of Hong Kong, Hong Kong; d Key Laboratory of Birth Defects and Related Diseases of Women and Children, Sichuan University, Ministry of Education, Chengdu, Sichuan Province, P.R. China; e College of Engineering, The Ohio State University, Columbus, OH.

**Keywords:** environmental endocrine disruptors, glucuronosyltransferases, polycystic ovary syndrome, single nucleotide polymorphism

## Abstract

**Background:**

In recent years, there has been an interest in whether environmental endocrine disruptors (EEDs) may contribute to the endocrine disorders in patients with polycystic ovary syndrome (PCOS). The clearance of EEDs from the human body is regulated by the glucuronidation of UDP-glucuronosyltransferases (UGT). This study aimed to analyze the relationship of *UGT1A1*, *UGT2B7*, and *UGT2B15* polymorphisms with the metabolism of EEDs in patients with PCOS.

**Methods:**

A total of 357 Chinese women (119 PCOS cases and 238 controls) were genotyped for polymorphisms of *UGT1A1*^*G71R*^, *UGT2B7*^*H268Y*^, and *UGT2B15*^*D85Y*^. The plasma concentrations of EEDs were measured by the gas chromatography-mass spectrometry method. The association between UGT polymorphisms and the serum level of EEDs in patients with PCOS was analyzed.

**Results:**

The *UGT2B7*^*H268Y*^ single nucleotide polymorphism was associated with an increased risk of PCOS. The homozygous polymorphism (TT) of *UGT2B7*^*H268Y*^ showed higher bisphenol A and PAEs concentrations in serum. However, a single nucleotide polymorphism on *UGT2B15*^*D85Y*^ expression was associated with a decreased risk of PCOS. Subjects homozygous for the T allele of *UGT2B15*^*D85Y*^ had a significant effect on phthalates in the blood. In addition, our results also showed that the homozygous polymorphism (TT) of *UGT2B7*^*H268Y*^ and *UGT2B15*^*D85Y*^ was associated with the capacity of the excretion of androgen in patients with PCOS.

**Conclusions:**

Our study reported the novel associations between the UGT polymorphisms and EEDs concentrations in patients with PCOS, supporting the relevance of genetic differences in EEDs metabolism, which might be considered as an etiology of PCOS.

## 1 Introduction

Polycystic ovary syndrome (PCOS) is a heterogeneous disorder characterized by multiple endocrine disruptions, and its underlying causes, although uncertain, are likely to be both genetic and environmental.^[1]^ Environmental endocrine disruptors (EEDs), such as bisphenol A (BPA), polybrominated diphenyl ethers (PBDEs), and phthalates (PAEs), are exogenous substances that interfere with hormone biosynthesis and metabolism and cause deviations from normal homeostatic control and reproduction.^[2]^ The “environmental” contributors that have been considered are related to obesity and lifestyle, and indeed, there is extensive evidence that both play important roles in PCOS.^[3]^ Some previous studies reported that women with PCOS had higher serum BPA concentration in comparison to normal healthy control.^[1]^ The women who exposed to BPA had higher BPA level and higher luteinizing hormone ratio follicle-stimulating hormone ratio than healthy women without BPA contact, and these observations confirmed the potential role of BPA in PCOS pathophysiology.^[4]^ Endocrine disruption by EEDs in humans may act via 2 main pathways: through hormone receptors (estrogenic and androgenic receptors and G protein–coupled estrogen receptor) activating or inhibiting gene expression^[5]^ and by altering the synthesis or degradation of the hormones naturally present in the organisms (i.e., aryl hydrocarbon receptor).^[6]^ Peretz et al^[7]^ reported dose-dependent and time-dependent decreases in estradiol (E2), estrone, testosterone (T), androstenedione, and dehydroepiandrosterone synthesis after 12 hours of exposure to 100 and 10 μg/mL BPA. These doses of BPA also compromised follicle growth. Finally, the EEDs can disrupt ovarian function, androgen activity, and metabolic regulation, which contribute to the etiology of PCOS.

UDP-glucuronosyltransferase (UGT) is a major phase II enzyme of the detoxification system which catalyses the conjugation of nucleophiles with glucuronic acid from the cofactor UDP-glucuronic acid. After the glucuronidation reaction, the hydrophilicity of compounds increases, contributing to its excretion by the biliary or urinary route,^[8–10]^ which are the most important pathways for the human body's elimination of endogenous and exogenous chemicals. It is known that the glucuronidation of UGT is a primary metabolizer for the clearance of EEDs from the human body. Similarly, the glucuronidation of UGT is also the major route for androgen inactivation and excretion.^[11]^ Many previous studies showed that the polymorphisms of UGT might affect its functions. For instance, the polymorphism of *UGT1A1*^*G71R*^ (G>A, rs4148323) is the most concerning for irinotecan-based regimens in non–small cell lung cancer, and is regarded as a predictor for severe adverse events. Nie et al^[12]^ indicated that *UGT1A1∗*6 and *UGT1A1∗28* polymorphisms reduced the activity of *UGT1A1* by as much as 40% to 60%. A frequent polymorphism of the *UGT2B7*^*H268Y*^ (C>T, rs7439366) has shown a variable functional impact with different substrates. Levesque et al^[13,14]^ suggested that the TT genotype of *UGT2B15*^*D85Y*^ (G>T, rs1902023) was twice as active as the GG genotype in the glucuronidation of dihydrotestosterone, which led to better protection against high androgen levels and decreased the risk of prostate cancer.^[15]^ To our knowledge, the glucuronidation of BPA is mainly mediated by *UGT2B15*, with other UGTs (*UGT1A1*, *UGT1A3*, *UGT1A9*, *UGT2B*4, and *UGT2B7*) contributing to a lesser extent.^[16]^ However, to date, the glucuronidation of PBDEs and PAEs is unclear.

Among clinical patients, most of them failed to provide an obvious exposure history to EEDs, with a few exceptions. Their EEDs concentrations in serum varied greatly, as they lived in similar environments. The great changes may have been influenced by individual internal factors, such as the differences in the functional activity of UGT caused by the polymorphism of genes. Taken together, we aimed to investigate the functional *UGT1A*1, *UGT2B7*, and *UGT2B15* genetic polymorphisms in 357 Chinese women, and thus to assess the effects of UGT single nucleotide polymorphisms (SNPs) on EED metabolites in patients with PCOS.

## 2 Materials and methods

### 2.1 Study participants

Three hundred fifty-seven women (119 PCOS cases and 238 controls) from 2014 to 2016, aged 12 to 44 years, who had lived in Chengdu for at least 6 months, who had menses for at least 2 years, were included in the present study. Participants who had used hormones in the previous 3 months were excluded. Pregnant women were also excluded. The study was approved by the Human Ethics Committee of West China Second University Hospital. The diagnosis of PCOS was based on the Rotterdam criteria, with the association of at least 2 of the 3 following criteria: oligo-ovulation or anovulation evidenced by oligomenorrhea (8 or less menstrual cycles in the preceding year) or the serum progesterone level in the luteal phase (<5 ng/mL); clinical and/or biochemical signs of hyperandrogenism (HA); and polycystic ovaries.^[17]^ Congenital adrenal hyperplasia, androgen secreting tumors, Cushing syndrome, thyroid disease, Gilbert syndrome, Crigler-Najjar syndrome, and prolactinoma were excluded. The 119 women with PCOS diagnosed according to Rotterdam recommendations were divided into 4 different phenotypes: group A, defined as having oligo-ovulation (O), HA, and polycystic ovary (PCO), was the most common phenotype seen in 64.5%; group B, defined as having O and HA, was seen in 7.5% of the phenotype; group C, defined as having HA and PCO, was seen in 14.3% and group D, defined as having O and PCO, was seen in 13.7%.

Controls matched for age were randomly chosen from the non-PCOS participants of case-control investigation with the ratio of 1:2. The authors had access to information that could identify individual participants during or after data collection.

### 2.2 Detection of EEDs

Fasting blood samples (15 mL) were collected from all subjects. All samples were taken during the early follicular phase of the menstrual cycle (between the third and seventh days) or after at least 3 months of amenorrhea. Blood samples were centrifuged immediately. The plasma was separated and stored at −80°C until assay. BPA, PBDEs, and PAEs were measured by the gas chromatography-mass spectrometry method, and the following instruments were used for the experiments: Trace Dynamax spectrum of quadrupole gas chromatograph-mass spectrometer (Thermo Fisher Corp), nitrogen blowing instrument (ANPEL Laboratory Technologies, Shanghai,China), Laborota 400 efficient rotary evaporator (Heidolph Corp, Germany), and SB 3200DT ultrasonic cleaner. Hexane, dichloride methane, and methanol of high-performance liquid chromatography grade were purchased from BCR Company.

### 2.3 DNA extraction and genotyping

Genomic DNA was extracted from peripheral blood using the phenol/chloroform method. The polymorphisms of *UGT 1A1*^*G71R*^ (rs4148323), *2B7*^*H268Y*^ (rs7439366), and *2B15*^*D85Y*^(rs1902023) were detected by polymerase chain reaction (PCR), and the direct sequencing PCR primers were synthesized by Tsingke (Chengdu, China). The following PCR primers and conditions were used *UGT1A1*^*G71R*^, 5’-AATGGATCCTGAGGTTCTGG-3’ and 5’-ATGAGCTCCTTGTTGTGCAG-3’ at 60°C annealing; *UGT2B7*^*H268Y*^, 5’-ATTCCTGTC AGGAAGACCCA -3’ and 5’-GTGTAAGTCAAACACTCTGAA -3’ at 58°C annealing; *UGT2B15*^*D85Y*^, 5’-ACCAGGATGTCTCTGAAATG -3’ and 5’-TGGTCCCA CTTCTTCAGATC -3’ at 58°C annealing. Direct sequencing was performed using a 3730xl DNA Analyzer and Sequencing Analysis 5.2 software.

### 2.4 Statistical analysis

We investigated associations between log_10_-transformed serum EEDs and UGT SNPs in PCOS patients. All the statistical analyses were completed using SPSS 18.0 software. Data were expressed as the mean (M) ± standard deviation or geometric mean ± geometric standard deviation. The χ^2^ test and odds ratio (OR) values were used to analyze the distribution of UGT genotypes in all subjects. One-way analysis of variance was used to investigate the association between serum concentrations of EEDs and androgen in different genotypes, an independent *t* test was used to investigate serum level of EEDs, and a value of *P* < .05 was considered statistically significant.

## 3 Results

### 3.1 The clinical characteristics of the study participants

One hundred nineteen PCOS cases participated in the present study. The general baseline levels between the case and control groups were comparable (Table 1).

**Table 1:**
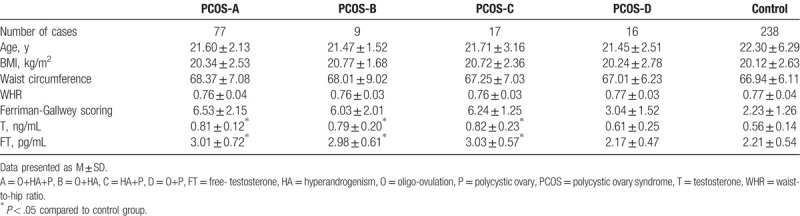
Background factors of the participants.

### 3.2 The genotype frequencies of UGT-related polymorphisms

The allele and genotype frequencies of the genotyping analysis are shown in Table 2. All the genotype frequencies were in Hardy-Weinberg equilibrium. The frequency of the G allele at the *1A1*^*G71R*^ site, C allele at the *2B7*^*H268Y*^ site, and major allele G at the 2B15^*D85Y*^ site was 74.5%, 70.5%, and 61.8%, respectively, which was consistent with other reports.^[18–20]^ Comparing the genotype distribution, the results showed that the TT genotype of *UGT2B7*^*H268Y*^ was associated with an increased risk of PCOS (OR = 1.87, 95% confidence interval = 1.02–3.43). In contrast, the TT genotype of *UGT2B15*^*D85Y*^ was associated with a decreased risk of PCOS (OR = 0.46, 95% confidence interval = 0.21–0.98). The distribution of *UGT1A1*^*G71R*^ showed no significant difference between the PCOS and control group (Table 2).

**Table 2:**
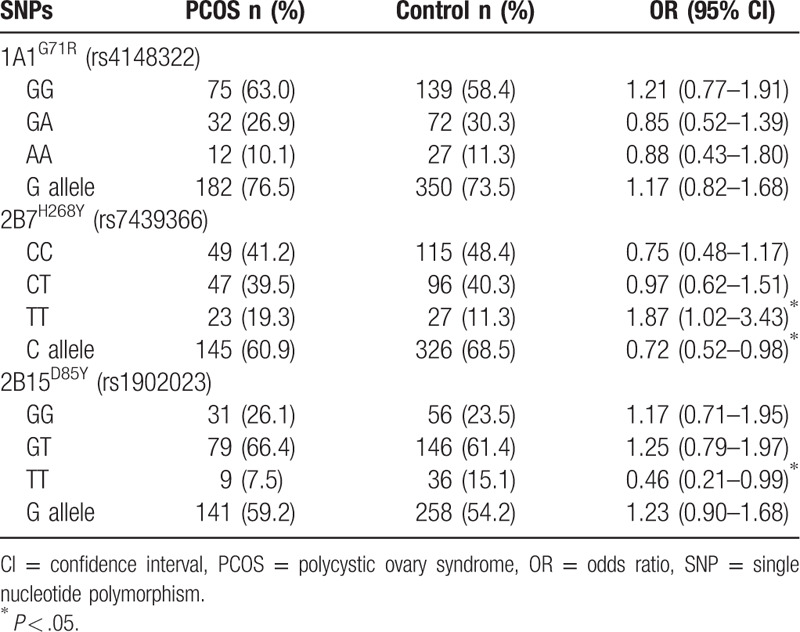
Distribution of *UGT1A1*, *UGT2B7*, and *UGT2B15* genotypes.

### 3.3 Detection and distribution of EEDs and androgen

Due to the limit of detection varying by the available sample volume for each sample, some samples were not detected. We summarized the detected ratio and the range of 3 kinds of EEDs (Table 3). Our results indicated that patients with PCOS had a higher BPA concentration (6.42 ± 3.74 ng/mL) when compared to the control group (4.76 ± 2.91 ng/mL, *P* = .01). Consistently, PAE level was also much higher in patients with PCOS (58.51 ± 36.45 mg/L) compared to the controls (41.46 ± 30.07 mg/L, *P* = .03) (Table 4). The PBDEs level in PCOS group was comparable with control group.

**Table 3:**
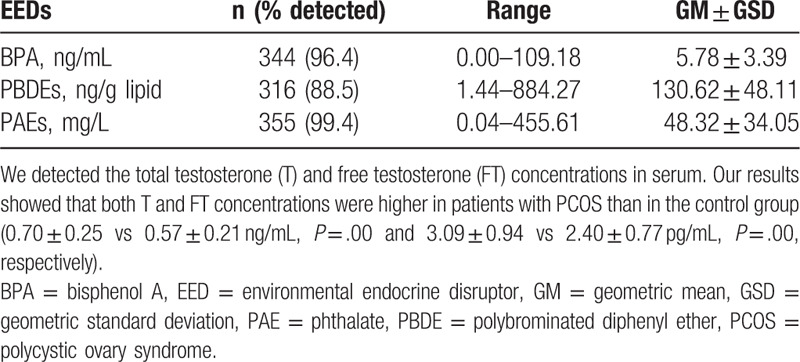
Detection and distribution of environmental endocrine disruptors.

**Table 4:**
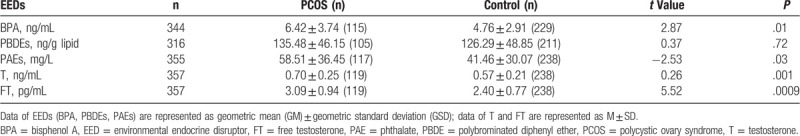
Distribution of environmental endocrine disruptors and androgens in polycystic ovary syndrome and control groups.

### 3.4 Effects of the *UGT1A1* polymorphism

**Figure 1:**
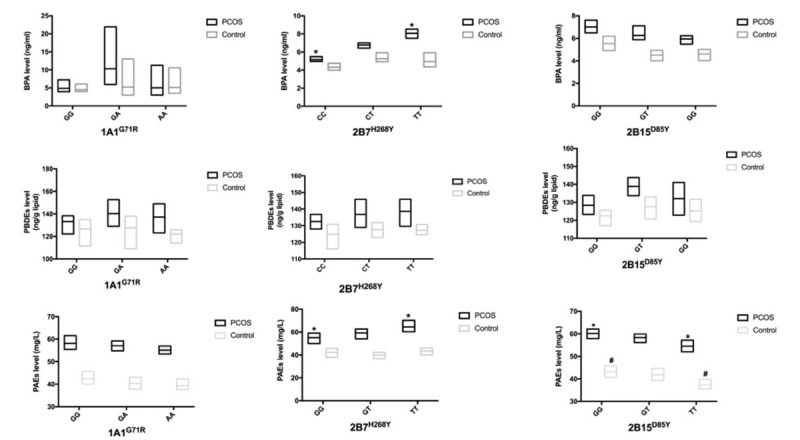
The association between glucuronosyltransferase (UGT) polymorphisms and environmental endocrine disruptors (EEDs) levels. Hyperandrogenemia (HA). ^∗^, ^#^*P* < .05 between the 2 groups, respectively. Data are represented as geometric mean (GM) ± geometric standard deviation (GSD). BPA = bisphenol A, PAE = phthalate, PBDE = polybrominated diphenyl ether, PCOS = polycystic ovary syndrome.

**Figure 2:**
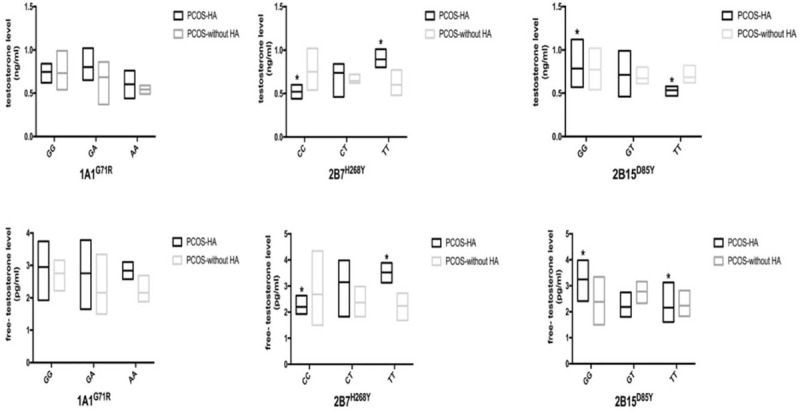
The association between glucuronosyltransferase (UGT) single nucleotide polymorphisms (SNPs) and T, FT levels. Testosterone (T), free testosterone (FT). Data are represented as M ± SD, ^∗^*P* < .05. HA = hyperandrogenemia, PCOS = polycystic ovary syndrome.

In this work, the distribution of *UGT1A1*^*G71R*^ in patients with PCOS was similar to that in the control group (Table 2). Furthermore, we did not find that the polymorphism of *UGT1A1*^*G71R*^ was associated with either EEDs (Fig. 1) or androgen concentration (Fig. 2) in serum.

### 3.5 Effects of the *UGT2B7* polymorphism

A previous study showed that the TT genotype was associated with decreased glucuronidation activity.^[21]^ In the present study, the results suggested that the *UGT2B7*^*H268Y*^ polymorphism had effects on the serum levels of EEDs and androgen. Patients with PCOS with the TT genotype had a higher BPA concentration (8.06 ± 2.01 ng/mL) when compared to the wild type (HH) (5.15 ± 2.91 ng/mL, *P* = .01) (Fig. 1). Moreover, in PCOS group, the PAEs concentration was also significantly higher in the TT group (64.56 ± 31.79 mg/L) when compared to the CC group (55.02 ± 33.25 mg/L, *P* = .01). There was no obvious association between the *UGT2B7*^*H268Y*^ SNP and PBDEs concentration.

In addition, the TT mutation in PCOS patients with hyperandrogenemia was related to the higher testosterone (0.91 ± 0.12 ng/mL) and free testosterone concentrations (3.47 ± 0.32 pg/mL) compared to the CC individuals (0.59 ± 0.20 ng/mL, *P* = .03 and 2.58 ± 0.61 pg/mL, *P* < .05, respectively) (Fig. 2).

### 3.6 Effects of *UGT2B15* polymorphism

The *UGT2B15*^*D85Y*^ polymorphism had an impact on the PAEs level. Both PCOS cases and controls, the TT genotype was significantly associated with a lower PAEs concentration (54.51 ± 35.93, 37.44 ± 28.57 mg/L) compared to the GG group (60.14 ± 34.72 mg/L, *P* = .01; 43.16 ± 33.73 mg/L, *P* = .02, respectively). Serum levels of BPA and PBDEs were not associated with the *UGT2B15*^*D85Y*^ polymorphism (Fig. 1). In addition, patients with PCOS with hyperandrogenemia also had a tendency toward lower testosterone (0.54 ± 0.18 ng/mL) and free testosterone (2.11 ± 0.60 pg/mL) levels in the TT group compared to GG group (0.75 ± 0.15 ng/mL, *P* < .05; 3.31 ± 0.72 pg/mL, *P* < .05, respectively) (Fig. 2).

### 3.7 Positive relationship between BPA and T serum concentration

To date, there is not a unified reference to divide BPA into high or low levels in the human body.^[4,22–24]^ In this work, we used the 75th percentile (P75) value to divide the subjects into either a hyper-BPA (>P75) or a hypo-BPA (≤P75) group. Our results suggested that the subjects in the hyper-BPA (>P75) group also had higher T concentration (*t* = 2.34, *P* = .03) (Table 5).

**Table 5:**
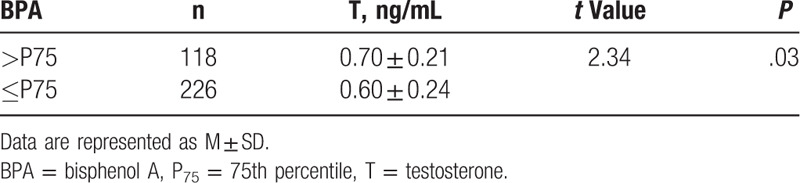
The relationship between bisphenol A and T serum concentration.

## 4 Discussion

To our knowledge, this is the first report in which the altered glucuronidation capability of EEDs has been attributed to the UGT SNPs in patients with PCOS. The frequencies of polymorphisms of *UGT1A1*^*G71R*^, *UGT2B7*^*H268Y*^, and *UGT2B15*^*D85Y*^ from 357 subjects here were consistent with other reports in Chinese or Asian people. In the present study, the results indicated that the TT genotype of *UGT2B7*^*H268Y*^ was associated with an increased risk of PCOS, through a decreased clearance of EEDs (BPA, PAEs) and androgen (T, FT) in patients with PCOS. In addition, the homozygous (TT) mutation of *UGT2B15*^*D85Y*^ was associated with a decreased risk of PCOS, via promoting the removal of PAEs and androgen (T, FT) in serum. These results suggested that these SNPs of UGT might affect its functions, presumably by decreasing/increasing the efficiency of EEDs clearance in patients with PCOS.

It is known that the glucuronidation of UGT is a primary metabolizer for the clearance of EEDs from the human body. BPA undergoes hepatic first-pass metabolism to an extent of approximately 90% and is converted to phase II metabolites, which are excreted in the urine.^[25]^ The glucuronidation of BPA is mainly mediated by *UGT2B15*, with other UGTs (*UGT1A1*, *UGT1A3*, *UGT1A9*, *UGT2B4*, and *UGT2B7*) contributing to a lesser extent.^[16]^ Previous studies reported that a mutation in *UGT2B7*^*H268Y*^ might significantly decrease the glucuronidation activity of the enzyme.^[21,26]^ Consistently, our results showed that the TT mutation of *UGT2B7*^*H286Y*^ induced a decreased clearance of BPA, resulting in a higher level of serum BPA in PCOS. Previous study suggested a positive relationship between BPA level and LH: FSH ratio in seller women, which confirmed the potential effects of BPA in PCOS etiology.^[4]^ Many studies have reported the effects of *UGT2B15* SNPs in metabolism; however, the results are inconsistent. Hanioka et al^[27]^ reported that D85Y substitution in *UGT2B15* decreased enzymatic function in vitro, which was closely associated with variations in the metabolism and toxicity of BPA. Levesque et al,^[13]^ however, found that the TT genotype of *UGT2B15*^*D85Y*^ increased the glucuronidation activity of the enzyme in HK293 cells, compared to the GG genotype. In this study, our results suggested that the TT genotype of *UGT2B15*^*D85Y*^ was more active than the GG genotype in the glucuronidation of PAEs. The discrepant results may in part account for the more complicated metabolic pathway in vivo, and the different approaches to extracting and measuring EEDs in human biospecimens may be because the sensitivities, specificities, and reliabilities of analytical methods vary across laboratories.^[28]^ It was reported that the toxic effects of PBDEs were of concern due to hydroxylated PBDEs (OH-PBDEs).^[29]^ Our study examined the association between PBDEs, not hydroxylated PBDEs, with UGT SNPs, and there was no association between UGT polymorphisms and the serum level of PBDEs. UGT is a superfamily, and our results may imply that PBDEs are not the direct substrate for these 3 kinds of UGTs.

The hormonal anomalies characteristic of PCOS include androgen excess and insulin resistance. It is now well established that *UGT2B7*, *UGT2B15*, and *UGT2B17* are the 3 major enzymes responsible for the glucuronidation of all androgen and their metabolites in humans.^[30]^ Our results suggested that patients with PCOS had higher T and FT concentrations than those in the control group, which could be explained by the following 2 potential causes: firstly, the TT mutation of *UGT2B7*^*H268Y*^ decreased the clearance of androgen in PCOS; secondly, although the TT mutation of *UGT2B15*^*D85Y*^ promoted the excretion of androgen, the TT mutation frequency was much lower in patients with PCOS. In addition, Takeuchi et al^[31]^ found that androgen level was correlated with BPA level in women. The relationships between androgen and BPA are unlikely to be simple, causal one. One possibility is that by binding to sex hormone–binding globulin, BPA displaces a proportion of the bound androgen, leading to higher free levels of androgen.^[32]^ Another possibility is that BPA interferes with androgen catabolism. Indeed, in rat liver, BPA administration reduces the levels of enzymes needed for testosterone hydroxylation.^[33]^ In this work, the results showed that the T concentration was also higher in the hyper-BPA (>P75) group (0.70 ± 0.21 ng/mL) compared to the hypo-BPA group (0.60 ± 0.24 ng/mL). This result showed that the clearance of androgen is negatively related concomitantly with the elevation of BPA level in serum. This result also implied a possible competitive binding between BPA, T, and UGT, which might be the other potential cause of androgen excess in patients with PCOS. Therefore, the clearance of androgen might be associated with *UGT2B7* SNPs and BPA level in patients with PCOS, and whichever plays the dominant role requires further study.

In our study, the *UGT1A1*^*G71R*^ SNP was not associated with EED metabolites in patients with PCOS, which may partly be due to the small sample size. In previous studies, *UGT1A1* was mostly associated with drug glucuronidation,^[34,35]^ and Gramec Skledar et al^[9]^ reported that *UGT1A1* influences the glucuronidation of bisphenol S. This may indicate that *UGT1A1*^*G71R*^ is not the main enzyme for the removal of these 3 kinds of EEDs (BPA, PBDEs, PAEs).

## 5 Conclusions

In this case-control study of Chinese patients with PCOS, our results indicated that they were likely to be loaded with higher BPA and PAEs concentrations. It seems that the clearance of BPA and PAEs was associated with *UGT2B7*^*H268Y*^ and *UGT2B15*^*D85Y*^ polymorphisms. The excretion of androgen was associated with the polymorphisms of *UGT2B7*^*H268Y*^ and *UGT2B15*^*D85Y*^, and the BPA level in serum. Taken together, our data suggested that the clearance of EEDs from the human body, which is related to the homozygous variants of *UGT2B7*^*H268Y*^ and *UGT2B15*^*D85Y*^, might be considered as an etiology of PCOS.

## Author contributions

**Data curation:** Yunyao Luo.

**Methodology:** Ying Nie.

**Resources:** Yunyao Luo.

**Software:** Lu Tang.

**Supervision:** Liangzhi Xu.

**Writing – original draft:** Yunyao Luo.

**Writing – review and editing:** Charles C Xu.
